# Measuring the Quality of Life of Visually Impaired Children: First Stage Psychometric Evaluation of the Novel VQoL_CYP Instrument

**DOI:** 10.1371/journal.pone.0146225

**Published:** 2016-02-26

**Authors:** Valerija Tadić, Andrew Cooper, Phillippa Cumberland, Gillian Lewando-Hundt, Jugnoo S. Rahi

**Affiliations:** 1 Population, Policy and Practice Programme, Life Course Epidemiology and Biostatistics Section, University College London (UCL) Institute of Child Health, London, United Kingdom (United Kingdom); 2 Department of Psychology, Goldsmiths, University of London, London, United Kingdom; 3 Warwick Medical School, University of Warwick, Coventry, United Kingdom; 4 National Institute for Health Research (NIHR) Biomedical Research Centre at Moorfields Eye Hospital NHS Foundation Trust and UCL Institute of Ophthalmology, London, United Kingdom; 5 Great Ormond Street Hospital for Children NHS Foundation Trust, London, United Kingdom; 6 Ulverscroft Vision Research Group, London, United Kingdom; Sun Yat-sen University, CHINA

## Abstract

**Purpose:**

To report piloting and initial validation of the VQoL_CYP, a novel age-appropriate vision-related quality of life (VQoL) instrument for self-reporting by children with visual impairment (VI).

**Methods:**

Participants were a random patient sample of children with VI aged 10–15 years. 69 patients, drawn from patient databases at Great Ormond Street Hospital and Moorfields Eye Hospital, United Kingdom, participated in piloting of the draft 47-item VQoL instrument, which enabled preliminary item reduction. Subsequent administration of the instrument, alongside functional vision (FV) and generic health-related quality of life (HRQoL) self-report measures, to 101 children with VI comprising a nationally representative sample enabled further item reduction and evaluation of psychometric properties using Rasch analysis. Construct validity was assessed through Pearson correlation coefficients.

**Results:**

Item reduction through piloting (8 items removed for skewness and individual item response pattern) and validation (1 item removed for skewness and 3 for misfit in Rasch) produced a 35-item scale, with fit values within acceptable limits, no notable differential item functioning, good measurement precision, ordered response categories and acceptable targeting in Rasch. The VQoL_CYP showed good construct validity, correlating strongly with HRQoL scores, moderately with FV scores but not with acuity.

**Conclusions:**

Robust child-appropriate self-report VQoL measures for children with VI are necessary for understanding the broader impacts of living with a visual disability, distinguishing these from limited functioning per se. Future planned use in larger patient samples will allow further psychometric development of the VQoL_CYP as an adjunct to objective outcomes assessment.

## Introduction

The prevailing emphasis on patient-led assessment of the impact of disease and healthcare [1,2] has led to generic and disease-specific patient-reported outcome measures (PROMs) assessing health-related quality of life (HRQoL) in children [3,4]. HRQoL is recognised to be a complex construct requiring capture of the subjective perspective of the impact of a disease on the person in their social and cultural context [5]. However, reliable and valid self-report PROMs, which are designed specifically for use with children with visual impairment (VI) and which are grounded in their own perspectives of the impact of living with VI on quality of life (QoL) have been lacking [6]. This is not surprising given the absence of a conceptual framework and the scientific challenges of conducting research with a clinically complex and a numerically small population [7].

Existing generic measures of HRQoL have not been developed for visually impaired children, nor with them, in mind. Thus they lack items relevant to their lives and have low content validity with this population. Recognising the need for a measure of vision-related quality of life (VQoL) for children with VI, we recently reported our child-centred approach to item generation for a self-report VQoL instrument for children aged 10–15 years with VI and/or blindness (the VQoL_CYP) [8]. Development of the draft instrument was firmly grounded in a child-centred methodology, including individual semi-structured interviews with children to identify the issues relevant to their lives and consulting children on drafting and formatting the instrument [8,9]. In keeping with the ‘self-discrepancy’ theory of QoL [10,11], we aimed to explore the feasibility of an instrument that formally captured the gap between the visually impaired child’s current experiences and expectations (Actual vs. Ideal Status).

Here, we report on the first stage psychometric evaluation of the VQoL_CYP instrument. This includes piloting and initial validation, including concurrent construct validity evaluation with our recent Functional Vision Questionnaire for Children and Young People with Visual Impairment (FVQ_CYP) [12] as well as a generic HRQoL instrument. We delineated *a priori* issues relating to VQoL from those relating to functional vision (FV), in keeping with the World Health Organisation’s conceptual framework on disability [13] and the VQoL_CYP was developed in parallel to our FVQ_CYP, an independent complementary PROM enabling the child’s own assessment of their ability to perform vision-dependent tasks [12]. The development of these two distinct vision-related instruments addresses the frequent construct conflation between the concepts of VQoL, FV and visual function (VF) in the ophthalmic literature [6,14].

## Materials and Methods

### Patient identification and recruitment

Children were eligible if *i)* they were visually impaired, severely visually impaired or blind (visual acuity [VA] in the better eye of LogMAR [Logarithm of the Minimum Angle of Resolution] worse than 0.48) due to any disorder, but without any other significant impairment (i.e., learning, sensory, motor); and *ii)* they were aged 10–15 years. A stratified random subsample of children was invited to participate from a sampling frame comprising patients attending the Department of Ophthalmology and the Developmental Vision Clinic at GOSH, and the Paediatric Glaucoma Service and Genetic Eye Disease Service at Moorfields Eye Hospital, London UK. 69 participants for piloting (phase 1) and a further 52 for validation (phase 2) of the VQoL_CYP were drawn from these sources. 49 additional eligible patients for the validation phase were recruited from 14 additional hospitals in the UK.

### Ethical considerations

The family physician was contacted and informed of the aims and the design of the study. Participants gave individual informed written assent and their parents gave written consent to participation. In a small number of cases in which children could not give written assent due to being severely visually impaired or blind, the child gave an informed verbal assent and a parent signed the corresponding assent form on their child’s behalf. The study protocol and materials, including the consent and assent forms and procedures, were approved by the Research Ethics Committee for UCL Institute of Child Health and Great Ormond Street Hospital (GOSH), London, UK (REC reference: REF: 07/Q0508/61). The study followed the tenets of the Declaration of Helsinki.

After initial stages of instrument development [8], piloting and initial validation of the instrument were undertaken in 2 distinct phases, as described below.

### Phase 1: Piloting

Children were invited to participate through a postal survey. The posted materials included an invitation letter, information sheets for children and parents, consent and assent forms and large print and electronic (CD) versions of the pilot child self-report VQoL_CYP instrument and a prepaid postage envelope for return of completed materials. A feedback form requested information on the time taken to complete the VQoL_CYP, difficulty of questionnaire instructions and completion, if/what assistance had been required and preference for mode of administration. 11 of 69 recruited children requested one-to-one instrument administration at home.

The pilot VQoL_CYP [8] comprised 47 items, each presented as a vignette describing a VQoL issue from an ‘illustrative’ (using gender-appropriate name) child’s perspective (e.g. *‘Ben feels frustrated because of his eyesight*’). Each asked the respondent firstly how much s/he *is presently* like this (‘*How much are you like Ben*?’ i.e. ‘Actual Status’) and then how much s/he *wishes to be* like that child (*‘How much do you want to be like Ben*?’, i.e. ‘Ideal Status’) using 4 response options: ‘1 = not at all’, ‘2 = a little bit’, ‘3 = quite a lot’, and ‘4 = exactly’. To avoid respondent bias, 35 statements were framed positively and 12 negatively (with reversed scoring). The scale was designed to produce independent summary scores for Actual Status and Ideal Status components (by adding up the item scores on each scale), higher scores on each indicating ‘better’ VQoL and larger differences between the two indicating greater ‘self-discrepancy’.

#### Data screening

Data were entered into an Excel database, where independent review of a random 30% sample of entered questionnaires showed no errors.

#### Respondent burden

Using the feedback form we assessed mean time for and difficulty of completion and instructions (using a 4-point scale), proportion of responders fully self-reporting, the help received (i.e. reading, writing or understanding the questions) and preferred mode of administration (print, electronic, audio, Braille, in-person administration by a professional).

#### Preliminary item reduction

This was guided by the pattern of missing data (i.e. excluding items with >50% of missing data as they are likely ambiguous and irrelevant to a large proportion of respondents [15]), distribution of the individual item responses (i.e. skewness and kurtosis within -2.00 and +2.00 bands, skew to ceiling/floor effect if >60% responses in an item end category) and item-total correlations (acceptable criterion *r* >.3) [15].

### Phase 2: Validation

To assess construct validity of the draft VQoL_CYP instrument, we concurrently administered our FV instrument (FVQ_CYP) [12] and a generic HRQoL instrument, the Pediatric Quality of Life Inventory (PedsQL) [16] via a postal survey. The FVQ_CYP captures the child’s self-reported level of difficulty in performing tasks or activities for which vision is required on 36 items and a 4-point scale. Higher total summary scores indicate greater difficulty. The scale has good psychometric properties [12]. The PedsQL [16] is a widely used 23-item questionnaire assessing generic HRQoL in children and young people 3–18 years across 4 domains (Physical, Emotional, Social and School Functioning). Total Scale Score, as well as Physical Health and Psychosocial Health summary scores are calculated, higher scores indicating better HRQoL.

Eligible families received a study pack as in the piloting phase. The large print child self-report versions of the 3 instruments were stapled together in a randomly assigned order.

#### Data screening

Data were entered into an Excel database with double data entry of 16% of questionnaires to identify and correct errors and independent data checking of the remainder.

#### Psychometric analyses

As the first step towards formal psychometric evaluation of the VQoL_CYP, item reduction was conducted using Rasch analysis in Winsteps (version 3.75.0) [17]. Rasch analysis [18] is a probabilistic mathematical model. It is based on the assumption that the probability that a person will endorse a particular response category in a scale item is a logistic function of the difference between the person’s characteristics (e.g. ability) and the item characteristics (e.g. difficulty). This allows for item and person parameter estimates to be calibrated on the same latent interval scale, expressed in logits (logarithm of the odds units). As all VQoL_CYP items have the same format and use the same categorical rating scale we applied the Andrich Rating Scale Model [19].

In line with the extant literature on the development of Rasch-calibrated rating scales [15,19] we assessed the following criteria:

1. *Item fit*. This was investigated by examining item infit and outfit statistics, which indicate how well the items fit the underlying construct (i.e. VQoL in this instance). Mean square standardized residuals (MNSQ), with 0.5–1.5 range are considered acceptable for productive measurement [20];
2. *Differential item functioning (DIF)*. This shows whether subgroups of participants with the same ‘ability’ in fact respond differently to items. We examined DIF by the key demographic variables age and gender, in keeping with other similar studies [21], as these variables should not differentially impact on how children respond to individual items. Valid comparison of the impact of living with visual disability on children across age (split into 10–12 and 13–15 age groups, due to the modest sample size) and gender groups requires the novel VQoL_CYP measure to be comparable and invariant across these groups and any notable DIF (standard threshold> 1.0 logit for notable DIF [22]) on these variables would confound subsequent comparison on these variables [23];
3. *Response scale ordering*. This was done by examining Rasch category probability curves, to demonstrate the likelihood of each response category on our 4-point scale being selected over the range of the scale [15];
4. *Targeting*. This was done by examining the item-person map, which illustrates a relative position of ‘item difficulty’ to ‘person ability’ (difference of person and item means of up to 1 logit is considered acceptable [22]);
5. *Measurement precision*. This refers to the ability of the instrument to discriminate between different groups of respondents on the measured variable and is examined by observing the person separation index and reliability indices (≥2.00 and >.80 the minimum accepted levels respectively [15]).

Thereafter, we used multiple-pattern regression-based imputation [24,25] using SPSS (version 21) [26] to replace missing data before deriving summary scores.

Construct validity was examined in SPSS by comparing Pearson correlation coefficients (*r*) between VQoL_CYP scores and PedsQL and FVQ_CYP scores as well as children’s VA and using the criteria by Pesudovs et al. [15] for determining convergent validity (i.e. the notion by which a measure is expected to correlate with another test measuring the same construct). By this criteria *r* range of .3-.9 is indicative of convergent validity with correlations *r* range = .3-.5 being considered ‘moderate’ and those with *r* > .5 being considered ‘strong’. Correlations with *r* < .3 are considered weak, thus failing the criteria for convergent validity, whereas correlations with *r* > .9 are considered too high, which would be indicative of a measure failing to provide significant additional information [15]. In order to take into account multiple correlations a more conservative alpha level of < .001 was set as the cut-off for determining statistically significant correlations.

## Results

Participation rates in both pilot and validation phases varied with a pooled estimate of 26%. This is comparable to similar studies [8,27] but notably this issue is rarely assessed or reported in the QoL literature [28]. Importantly, the patient samples achieved for both phases were unbiased, being representative of the overall UK population of children with VI or blindness without additional impairments [7] with respect to both demographic and clinical characteristics (Table 1).

**Table 1:** Clinical and socio-demographic characteristics of participants in piloting and validation phases.

	*Piloting phase*	*Validation phase*
*Participant characteristics*	*N (%) Total 69*	*N (%) Total 95*
**Age group**		
10–12 years	30 (43.5%)	59 (62%)
13–15 years	39 (56.5%)	36 (38%)
**Gender**		
Boys	36 (52.2%)	55 (58%)
Girls	33 (47.8%)	40 (42%)
Vision level*		
***Visual impairment (VI) LogMAR 0*.*50–1*.*00***		
VI 1: LogMAR 0.50–0.70	36 (52.2%)	43 (45.3%)
VI 2: LogMAR 0.71–1.00	25 (36.2%)	32 (33.7%)
***Severe visual impairment (SVI) and blindness LogMAR 1*.*01 or worse***		
SVI: LogMAR 1.01–1.30	4 (5.8%)	10 (10.5%)
Blind: LogMAR 1.31 or worse	4 (5.8%)	10 (10.5%)
**Course of visual loss**		
Stable	43 (62%)	53 (56%)
Progressive	26 (38%)	42 (44%)
**Timing of VI onset**		
Early (≤2 years)	61 (88%)	68 (71.6%)
Late	8 (12%)	27 (28.4%)
Diagnosis by site of VI**		
Whole globe and anterior segment	9 (13%)	2 (2.1%)
Glaucoma—primary or secondary	9 (13%)	8 (8.4%)
Cornea (sclerocornea and corneal opacities)	2 (2.9%)	3 (3.2%)
Lens (cataract and aphakia)	9 (13%)	9 (9.5%)
Uvea	3 (4.3%)	5 (5.3%)
Retina	37 (53.6%)	62 (65.3%)
Optic nerve	8 (11.6%)	10 (10.5%)
Cerebral/visual pathways	6 (8.7%)	4 (4.2%)
Other (idiopathic nystagmus, high refractive error)	7 (10.1%)	10 (10.5%)
Ethnicity***		
White majority	55 (82%)	78 (82%)
Minority (Asian, Black, Mixed, Other non-White)	12 (18%)	17 (18%)
Index of Multiple Deprivation****		
1: Most Deprived	7 (10.3%)	20 (22%)
2	10 (14.7%)	11 (12%)
3	14 (20.6%)	16 (17.6%)
4	14 (20.6%)	20 (22%)
5: Least Deprived	23 (33.8%)	24 (26.4%)

* World Health Organisation categories of visual impairment based on acuity in better seeing eye.

**Does not add up to 100% because some children had visual impairment originating in multiple sites. Data on 1 child is missing as diagnosis was not provided by the hospital where the patient was identified.

***Unknown for 2 children in piloting phase (i.e. not stated)

****Based on the UK postal code. Data on 1 child in piloting phase missing (child from Ireland). Data on 4 children in validation phase is missing as no postcodes were provided by the hospital where they were identified.

Acronyms: VI-visual impairment, SVI-severe visual impairment, LogMAR-the Logarithm of Minimum Angle of Resolution

Of 101 consenting children in the validation phase, 3 children were excluded having left the VQoL_CYP questionnaire blank, 2 did not meet the key eligibility criteria (one being 18 years of age and one having severe learning difficulties and additional impairments) and 1 was a duplicate child recruited in this phase from separate sources at the same time (with first assessment being considered for the subsequent analyses). Subsequent analyses in this phase were completed on 95 children.

### Phase 1: Piloting

#### Respondent burden

Mean completion time for the VQoL_CYP was 15.5 minutes (SD = 10.6, 12/69 children data unavailable), the maximum duration proposed for people with disabilities [29]. Completion time was not associated with age or severity of VI. Although over 85% of children rated the instructions and over 95% general questionnaire completion ‘easy’ or ‘very easy’, only 48% of all responders reported completing the questionnaire *completely* independently. Younger children (10–12 years) and those with severe VI or blindness (LogMAR acuity worse than 1.00) were more likely to report requiring *some* help (Pearson Chi Square *p* = 0.009 and *p* = 0.029 respectively). Where required, help was reported with reading the questionnaire (60% and 58% of younger and older children respectively), writing answers (53% and 43%), and fully understanding questions (63% and 67%). There was no significant difference in the VQoL_CYP summary scores between those who reported receiving help overall and those who did not. 58% of children reported their preferred option would be an electronic instrument, 35% print, 13% audio and 6% for Braille. Overall 41% would have preferred interviewer-assisted administration rather than self-report format, although preferences were not mutually exclusive, with most selecting more than one option.

#### Preliminary item reduction

The small amount of data missing at random (Actual Status <3%, Ideal Status <9%), did not warrant item removal (i.e. <50%). 4 items on the ‘Actual Status’ scale were highly skewed and 4 achieved item-total correlations below acceptable limits and were removed (see Appendix A in S1 File for all removed items). The remaining 39 items were administered in the validation phase. There was significant skewing of responses towards the ceiling effect (>60% responses in category 4 in 38 of 47 items) on the ‘Ideal Status’ scale. However, as this was an innovation intended to explore feasibility of quantifying ‘self-discrepancy’, for completeness we included the Ideal scale in the validation phase to confirm lack of feasibility as described below.

### Phase 2: Validation

#### Psychometric analyses

As anticipated from Phase 1, significant ceiling effect within the Ideal Status scale was observed again, confirming general redundancy of this as well as the ‘self-discrepancy’ scale, so we did not evaluate these 2 scales further. The Actual Status component was the sole index of VQoL thereafter for psychometric testing.

There was a low amount of data per item missing at random (≤3% on 18 of 39 items). Skewness and kurtosis were within the acceptable range. One item had over 60% responses in an end response category and was removed before Rasch analysis. 3 additional items were removed based on the Rasch fit statistics, which in the remaining 35 items were within the acceptable 0.5–1.5 limits (Table 2). Use of the conservative MNSQ range of .7–1.3 [15] to consider further item reduction could be re-considered when data from larger samples are available.

**Table 2:** Rasch fit statistics, item measure and DIF contrasts for the 35-item 4-point response VQoL-CYP instrument. Items are ordered by item measure (logits) from positive (items harder to endorse) to negative (items easier to endorse).

*Item code*	*Item*	*Item measure (logits)*	*Infit MNSQ*	*Outfit MNSQ*	*DIF contrast age (logits)*	*DIF contrast gender (logits)*
Q18	Is comfortable going to places on her/his own	0.81	1.34	1.36	0.79	0.6
Q29	Feels frustrated because of her/his eyesight	0.72	1.2	1.14	-0.22	-0.03
Q45	Has to work harder at school because of her/his eyesight	0.59	1.23	1.21	-0.18	-0.11
Q22	People overprotect her/him because of her/his eyesight	0.48	1.39	1.49	0.18	0.22
Q16	Feels different from other children and young people	0.47	0.83	0.81	-0.02	0
Q2	Makes new friends easily, despite her/his eyesight	0.33	1.01	0.96	-0.27	0.24
Q43	She/he can get around on her/his own	0.25	0.97	1.13	0.79	0.52
Q14	Her/his friends encourage her/him to join in their activities	0.21	1.11	1.15	-0.53	-0.43
Q13	Feels like he/she fits in	0.2	0.75	0.73	-0.07	-0.28
Q11	Her/his friends help her/him at school	0.18	1.22	1.29	-0.17	-0.52
Q40	Worries her/his eyesight will get worse	0.16	1.28	1.22	-0.54	-0.28
Q10	Gets treated the same as everyone else	0.13	0.78	0.76	0	0.03
Q24	Is given freedom to do things on his/her own	0.13	1.03	1.48	0.91	-0.16
Q25	Is comfortable asking for help	0.13	1.18	1.22	-0.39	0.32
Q44	Likes being at school	0.13	1.11	1.15	0.1	-0.72
Q36	Worries what other people think about her/his eyes	0.12	1.37	1.32	-0.48	0.17
Q6	Spends enough time with his/her friends	0.09	0.94	1.01	-0.13	-0.09
Q8	Can stand up for him/herself if someone picks on him/her	0.08	1.24	1.23	-0.51	0.56
Q9	His/her friends understand about his/her eyes	0.01	1.24	1.33	-0.13	-0.45
Q21	People give her/him chance to do things on his/her own	0	0.77	0.76	0.25	0.07
Q20	Can do most things his/her own	-0.02	0.87	0.88	0.77	0.1
Q32	Feels confident	-0.11	0.81	0.81	-0.46	0.18
Q5	Is happy with his/her social life	-0.13	0.77	0.74	-0.48	-0.08
Q17	In spite of her/his eyesight, she/he is independent	-0.13	0.85	0.86	0	-0.08
Q37	Is positive about future	-0.18	0.83	0.82	0.23	-0.05
Q38	Is confident she/he will be able to look after herself/himself when she/he is older	-0.18	0.87	0.85	0.52	0.39
Q15	Her/his teachers understand about her/his eyes	-0.26	0.91	0.95	-0.32	-0.24
Q31	Is treated fairly	-0.26	0.71	0.66	0.15	0
Q27	Copes well with his/her eyesight problems	-0.27	0.64	0.6	0.29	-0.05
Q28	Has enough time to her/himself	-0.3	0.89	0.86	0.43	0.37
Q12	Feels left out because of his/her eyesight	-0.47	0.98	0.98	-0.19	0.05
Q42	Likes to have a go at everything, despite her/his eyesight	-0.58	0.96	0.86	0.08	-0.3
Q30	Feels lonely because of her/his eyesight	-0.66	0.83	0.73	-0.03	0
Q3	Gets along with his/her family	-0.79	1.2	1.2	-0.26	0.06
Q1	Has got some good friends	-0.89	0.85	0.86	0.11	-0.08

Acronyms: DIF-differential item functioning; MNSQ—mean square; Q-question

**Fig 1:**
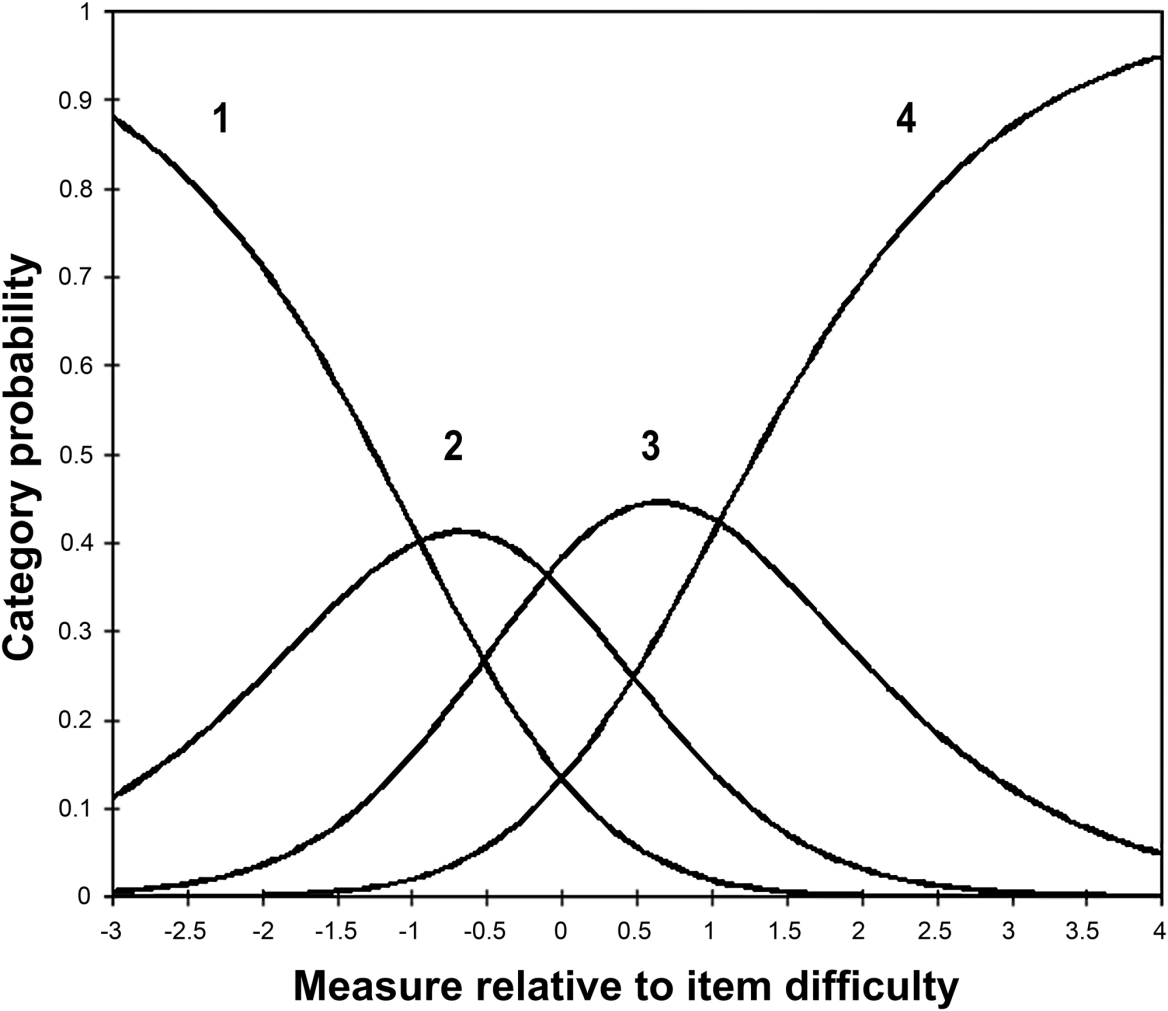
Category probability curves for 4 response categories, with a distinct peak for each category (1 = not at all, 2 = a little bit, 3 = quite a lot and 4 = exactly).

There was no notable DIF across either age group or gender (DIF contrasts<1.0 logit) (Table 2), although there was a tendency for a larger DIF for age on some items. The category probability curves were well ordered, with a clear separation between response categories and good coverage of the latent trait, supporting the four category rating scale (Fig 1). Good measurement precision was indicated by the person separation and reliability indices (2.67 and .88 respectively).

**Fig 2:**
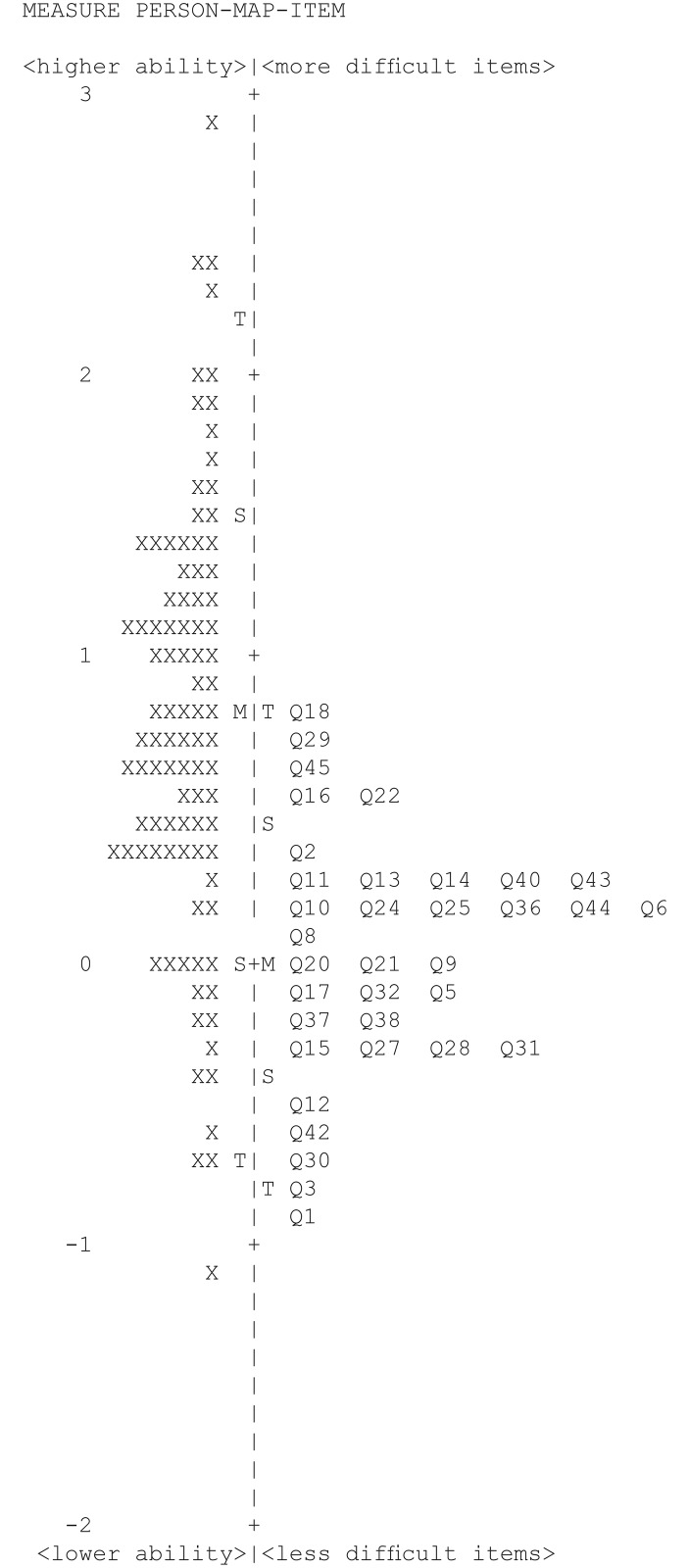
Person-item map illustrating targeting of items to participants. Items are located on the right of the dashed line. Participants are located on the left side of the dashed line and represented by X. Children with lower ‘ability’ and ‘less difficult’ items are at the bottom half of the map. M = Mean, S = 1 standard deviation from the mean, T = 2 standard deviations from the mean.

The person-item map (Fig 2) showed targeting of items to participants to be within acceptable limits (the difference between person and item means = -.77 logits), although with items clustering at the lower end of the item difficulty scale (i.e. items were easier to endorse relative to person ability).

Following multiple-pattern regression-based imputation of the small amount of missing data, summary scores for the reduced 35-item VQoL_CYP scale (with the 4 response categories recoded to 0–3 scale) and the FVQ_CYP and PedsQL were derived separately for the original dataset and individual and pooled imputation iterations.

Table 3 shows a strong correlation between VQoL_CYP and PedsQL Total Summary Score, especially its Psychosocial Health scale, together with a moderate negative correlation between VQoL_CYP and FVQ_CYP scores, demonstrating the construct and convergent validity of VQoL_CYP. The correlation of VQoL_CYP with the PedsQL Psychosocial summary score was of a significantly greater strength compared to the moderate correlation with PedsQL Physical Health summary score (Fisher’s r-to-z transformation test for difference between two correlation coefficients, *p* = 0.003) providing further evidence for strong psychosocial underpinning of the VQoL_CYP. Although VQoL_CYP and FVQ_CYP moderately correlated with each other, the VQoL_CYP scores did not correlate significantly with visual acuity. This suggests that VQoL is not necessarily related to the objectively measured severity of VI, in contrast to FVQ_CYP which correlated highly with acuity and which shares the functional disability aspect of VI with it (i.e. this providing convergent validity evidence for the FVQ_CYP [12]). The correlations between PedsQL components and acuity were weak (< .3) indicating divergence of HRQoL from acuity.

**Table 3:** Pearson coefficients for correlations across the outcome measures.

	1	2	3	4	5	6
1. VQoL_CYP Summary Score		-.502*	.664*	.738*	.455*	-.210
2. FVQ_CYP Summary Score			-.652*	-.599*	-.622*	.509*
3. PedsQL: Total Scale Score				.952*	.906*	-.263
4. PedsQL: Psychosocial Health Summary Score					.734*	-.220
5. PedsQL: Physical Health Summary score						-.279
6. Visual acuity						

*p < 0.001

Acronyms: VQoL_CYP -vision related quality of life instrument for children and young people; FVQ_CYP-functional vision questionnaire for children and young people; PedsQL-Pediatric Quality of Life Inventory

Limited statistical power of the study precluded conclusive investigation of further Rasch properties, such as unidimensionality. However, Cronbach’s alpha coefficient of the VQoL_CYP was 0.90, indicating high internal consistency of the VQoL_CYP. Similarly high Cronbach Alpha coefficients were found also for FVQ_CYP and PedsQL Total (0.97 and 0.89 respectively)

Details on instrument scoring and use are provided in Appendix B (in S1 File) and are obtainable from the corresponding author.

## Discussion

We report first stage psychometric evaluation of a novel age-appropriate PROM intended to capture self-reported VQoL of visually impaired children aged 10–15 years. Following piloting and initial validation, our current 35-item instrument has shown good psychometric properties. In the absence of a conceptual framework and established methodology for the development of child-appropriate vision-specific PROMs [6], we pursued a rigorous approach of recruiting children with VI aged 10–15 without any additional impairments, allowing capture of issues a) relevant to living with VI irrespective of additional diagnoses that may coincide with VI and b) in an age group that is well placed to self-report. Given the representative national sample, the novel VQoL_CYP should be applicable across the UK population of children with VI and/or blindness aged 10–15 years (cross-cultural validation is recommended for use outside the UK). This provides a robust template for further instrument testing and development.

The VQoL_CYP has high (and unique) content validity by virtue of being firmly and solely grounded in visually impaired children’s own perspectives of living with VI [8,9], unlike generic HRQoL measures for children like PedsQL. Additionally, it has strong construct and convergent validity, demonstrated through correlations with PedsQL, together with a negative correlation with FVQ_CYP, and lack of correlation with VA. The relatively strong, but not perfect, correlation with PedsQL and its psychosocial component in particular supports that the construct captured by the VQoL_CYP is indeed QoL rather than a functional outcome. Similarly, the moderate correlation of VQoL_CYP with FVQ_CYP is evidence of convergence that is expected between two PROMs designed to capture child-perceived vision-related impact of visual disability, but each in their unique ways (i.e. social-emotional consequences of living with VI in a societal context as opposed to its visual ability-based functional impact). This, combined with the lack of correlation with VA, confirms our *a priori* hypothesis that VQoL is distinct from functional ability [6] as well as providing unique information that is not available from objective clinical assessments. This is in keeping with the recent ophthalmic research with adults where existing instruments purportedly measuring VQoL have been re-calibrated and split into separate psychosocial and functional scales using Rasch analysis [30]. Thus, the VQoL_CYP shows promising value both as a stand-alone measure and a complementary adjunct to the FVQ_CYP and objective clinical assessments in routine clinical practice and research for comprehensive assessment of the impact of VI in childhood.

These positive and inverse correlations underpinning construct validity are in keeping with the ‘disability paradox’, exemplified by severely disabled or chronically ill people with significant functional limitations reporting good QoL [31]. Therefore, the interaction of multiple factors (e.g. personality, adjustment to the visual condition, family circumstances, professional support), rather than the severity of their VI per se, is likely to shape the visually impaired child’s VQoL. This illustrates both the complexity of QoL as a measurable theoretical construct that cannot be assessed solely by objective clinical parameters and the need for understanding children’s perceptions of their QoL without presumptions that are based on their clinical profiles alone.

Whilst we initially intended to explore whether ‘self-discrepancy’ could be formally captured using a dual ‘Actual and Ideal Status’ scale, we demonstrated that this was not feasible, although firmly advocated [11]. Most child QoL measures target the ‘current’ experience that forms the basis of our ‘Actual Status’ component, which has shown promise as a psychometrically valid VQoL index. Notably, removing the ‘Ideal Status’ also significantly reduces the respondent burden, making the instrument more practical for use in routine clinical practice.

It is worth emphasising that, although fully independent self-reporting by children is advocated as the gold standard, assessment and reporting of whether this is feasible is extremely rare. Thus, we specifically made an attempt to investigate this in our study and found a high percentage of participants required help with *some* aspect of instrument completion. As this was a postal survey, we were unable to fully ascertain the level of help received, even in those children who reported independent instrument completion. We recognise that this may have some bearing on the items. This issue is likely to be the case for many existing paediatric instruments but receives little attention. Although half of participants expressed a preference for electronic presentation, none completed the electronic (CD) version; thus, we assume the visual challenge of a print format was not a key factor. Over 40% children reported they would be happy to have the instrument researcher/clinician-administered, which is relevant to use in routine clinical practice. We suggest that administration by a professional would be preferable to parent-assisted completion to avoid parental influence on the child’s responses. Parental reports of their child’s VQoL (where obtainable concurrently using instruments with proxy as well as self-report versions), are valuable in their own right, especially to highlight meaningful divergence between parents’ and children’s views [32].

Our instrument seemed somewhat better targeted to children with low VQoL_CYP scores suggesting it may be particularly useful in assessing VQoL changes over time as a function of an intervention in children reporting low VQoL. This finding resonates with the targeting pattern of the IVI_C [21], a recent scale developed in Australia to assess VQoL of children and young people with VI aged 8–18 years.

The burden to participants in this phase of research prevented assessment of other important psychometric properties, such as temporal stability and responsiveness of the instrument over time through repeated assessments. The statistical power of this phase limited the possibility of conclusive formal evaluation of psychometric properties in general, including the extensive application of the Rasch analysis, including the evaluation of score dependency and principal component analysis of residuals to confirm unidimensionality. Given the high interest among clinicians and researchers who work with the complex, specific and numerically small group of children and young people with VI, and the knowledge of potential clinical relevance alongside the recognition of that this type of research is very challenging (requiring time to collect sufficient data to fine-tune the instrument), we have already made plans for future validation work with new patient samples in our collaborating clinical centres, where the instrument will be applied routinely. As well as addressing further psychometric evaluation, we will assess feasibility and effectiveness of the VQoL_CYP in routine practice (including acceptance by the different groups of patients and professionals and its potential role as an indicator of the quality of clinical provision). With increased patient samples, achieved through wider use of the instrument in clinical practices, it will be possible to re-visit item fit using more stringent criteria, unidimensionality, item dependency and further assessment of construct validity (e.g. in the context of trials and interventions). This will ultimately enable further item reduction, resulting in a more compact, precise and child user-friendly instrument. Furthermore, further research with larger samples planned by our group to adapt the VQoL_CYP also to younger children and older young people will allow for further detailed examination of any potential age or gender related DIF within the instrument that could potentially confound comparisons on these key variables.

In conclusion, our current VQoL_CYP instrument is a novel, age-appropriate instrument that shows good promise for use in evaluating the impact of living with a VI from the affected child’s perspective. Using such an instrument in routine clinical practice could provide a mechanism by which children can influence decisions about their clinical care and inform assessments of treatments, especially where changes in clinical parameters may be small. In its final version, the instrument could be used as a stand-alone measure or in conjunction with other child-relevant PROMs (e.g. our FVQ_CYP instrument) to contribute to improving the care and treatment of children with VI by providing information that is not captured by objective clinical outcome measures. The instrument’s use would potentially enhance clinicians’ understanding of the ‘real-life’ impact of VI on individual patients and how clinical care affects this, supporting a more ‘personalised’ approach to clinical care and decision-making for children with VI.

## Supporting Information

S1 File Appendices. (DOCX)
