# Supplementary material for: Measuring the Quality of Life of Visually Impaired Children: First Stage Psychometric Evaluation of the Novel VQoL_CYP Instrument
Source: PLoS One. 2016 Feb 26;11(2):e0146225. doi: 10.1371/journal.pone.0146225 (PMC4768881; doi:10.1371/journal.pone.0146225)
Supplement: S1 File — (DOCX) [file pone.0146225.s001.docx]

# Supporting information

**S1 file: Appendices**

**Appendix A: Removed items**

*Piloting:*

Items removed for skewness:

1. ‘is encouraged by family’
2. ‘has sighted friends’
3. ‘is used to living with VI’
4. ‘doesn’t let his/her VI stand in his/her way’

Items removed for individual response pattern:

1. ‘feels embarrassed about having extra help’
2. ‘feels braver because of living with VI’
3. ‘finds going to the eye clinic helpful’
4. ‘has a say in what happens to him/her at the eye clinic’

*Validation:*

Items removed for skewness (ceiling effect):

1. ‘gets picked on because of his/her eyesight’

Items removed due to fit statistics outside of the accepted limits:

1. ‘prefers friends with visual impairment’
2. ‘worries about what job s/he will be able to do in the future’
3. ‘has plans for the future’

**Appendix B: Instrument scoring and use**

The VQoL_CYP summary score is derived by converting the 1-4 ordinal responses across the 35 items to 0-3 scores and adding these up across the scale. A practical scoring sheet to facilitate this conversion can be obtained from the first author. Occurrence of missing data in survey research is common but it should not be ignored and the reasons for missing data should be explored. Although the multiple regression based imputation model of replacing missing data is a preferred method for research purposes, for practical use of the instrument (e.g. in clinical practice) a more conventional method of replacing the missing data by using the average of the rest of the items and thus deriving a ‘prorated’ scale score is acceptable, provided the percentage of missing data per person is within acceptable limits (see other paediatric literature [1,2]).

**References:**

1. Varni JW. Scaling and scoring of the Pediatric Quality of Life Inventory TM: PedsQL TM. 2014(28/04/2015). http://www.pedsql.org/PedsQL-Scoring.pdf.
2. Khadka J, Ryan B, Margrain TH, Court H, Woodhouse JM. Development of the 25-item Cardiff Visual Ability Questionnaire for Children (CVAQC). Br. J. Ophthalmol. 2010;94(6):730-735.
